# Features of the inflammatory response at the long-term stages of juvenile schizophrenia

**DOI:** 10.1192/j.eurpsy.2023.567

**Published:** 2023-07-19

**Authors:** S. A. Zozulya, S. A. Golubev, D. V. Tikhonov, V. G. Kaleda, T. P. Klyushnik

**Affiliations:** ^1^Laboratory of Neuroimmunology; ^2^Department of Youth Psychiatry, Mental Health Research Centre, Moscow, Russian Federation

## Abstract

**Introduction:**

Immunological study of late stages of schizophrenia manifesting in young adult age is of considerable interest for clarification of pathogenetic patterns of the disease and optimization of further treatment of patients.

**Objectives:**

To evaluate the relationship between the spectrum of inflammatory markers and psychopathological symptoms in patients with juvenile schizophrenia in a long-term follow-up study.

**Methods:**

34 patients with schizophrenia (F20) first manifested at the age of 16-25 years were followed-up for 20-25 years. The mean age of the patients at the time of follow-up study was 46.7±3.2 years. PANSS and PSP scales were used to quantify the severity of psychopathological symptoms. The control group consisted of 20 healthy people. Plasma immune parameters included leukocyte elastase (LE) and α1-proteinase inhibitor (α1-PI) activity, and antibodies to S100B and myelin basic protein.

**Results:**

Three types of juvenile schizophrenia follow-up outcomes were identified. The immunological heterogeneity of the types allowed us to distinguish groups of patients differing in the level of inflammatory activation. There were a significant increase in LE and α1-PI in patients of the first type (with a predominance of personality dynamics), a significant increase in α1-PI in patients of the second type (with actual negative disorders) compared to controls, and no significant differences with controls in LE and α1-PI in patients of the third type (with relevant positive and negative disorders).

**Conclusions:**

Residual psychopathological symptoms observed in the late stages of juvenile schizophrenia may be due to both low/moderate inflammation and genetic mechanisms.

**Disclosure of Interest:**

None Declared

